# Bedside practice of blood transfusion in a large teaching hospital in Uganda: An observational study

**DOI:** 10.4103/0973-6247.53872

**Published:** 2009-07

**Authors:** J. D. de Graaf, I. Kajja, G. S. Bimenya, M. J. Postma, C. Th. Sibinga

**Affiliations:** *Academic Institute IDTM, Groningen, Netherlands*; 1 *Department of Orthopaedics, Makerere University Medical School, Kampala, Uganda*; 2 *Department of Pathology, Makerere University Medical School, Kampala, Uganda*; 3 *Groningen Research Institute of Pharmacy (GRIP) University of Groningen, Groningen, The Netherlands*; 4 *Department of Epidemiology, University of Groningen, Groningen, The Netherlands*

**Keywords:** Blood transfusion, developing country, observational study, practice, transfusion reactions

## Abstract

**Background:**

Adverse transfusion reactions can cause morbidity and death to patients who receive a blood transfusion. Blood transfusion practice in Mulago Hospital, Kampala, Uganda is analyzed to see if and when these practices play a role in the morbidity and mortality of patients.

**Materials and Methods:**

An observational study on three wards of Mulago Hospital. Physicians, paramedics, nurses, medical students and nurse students were observed using two questionnaires. For comparison, a limited observational study was performed in the University Medical Centre Groningen (UMCG) in Groningen, The Netherlands.

**Results:**

In Mulago Hospital guidelines for blood transfusion practice were not easily available. Medical staff members work on individual professional levels. Students perform poorly due to inconsistency in their supervision. Documentation of blood transfusion in patient files is scarce. There is no immediate bedside observation, so transfusion reactions and obstructions in the blood transfusion flow are not observed.

**Conclusion:**

The poor blood transfusion practice is likely to play a role in the morbidity and mortality of patients who receive a blood transfusion. There is a need for a blood transfusion policy and current practical guidelines.

## Introduction

Blood transfusion is a tissue transplantation which can cause serious adverse reactions. These reactions may potentially delay the recovery of the patient or may even lead to death of patients. In Western countries, the frequency of adverse reactions which lead to major consequences for patients is relatively low. In 2006 in The Netherlands with a 16.5 million population, 578,145 units of blood were collected and 556,509 blood units were issued, where 1.2% of the patients suffered from severe morbidity and 0.2% of the patients died as a consequence of blood transfusion.[1]On the other hand, in Uganda with a population of 30 million people, out of the 150,000 units of blood collected annually, 135,000 units are administered. The remaining 15,000 units are discarded due to positive indicators of transfusion transmissible infections. Reports to show the various outcomes of the transfusions performed are hard to come by due to poor documentation.

There is limited information available on adverse transfusion reactions in developing countries. In many situations there is no proper and reliable documentation of the patient’s transfusion history. A preliminary observation of patients undergoing transfusion in the Makerere University teaching hospital Mulago in Kampala, Uganda, showed that the general condition of some patients deteriorates and others die after receiving a blood transfusion. However, it was not evident whether these changes in patient conditions were solely due to the transfusions or to the underlying disease processes. Worse, these events were not routinely recorded and analyzed for causal factors, which may include the blood transfusions per se.

We therefore analyzed the current bedside blood transfusion practices in Mulago Hospital, with an underlying hypothesis that the handling and dispensing of clinical transfusion procedures has a direct relationship to transfusion outcomes. To put our local observations into perspective a comparative analysis was performed at the University Medical Centre Groningen (UMCG), The Netherlands.

## Materials and Methods

The study was a SWOT (strengths, weaknesses, opportunities and threats) analysis on the basis of an observational study on transfused patients and their hospital environment, carried out in Mulago Hospital, Kampala, Uganda. This teaching hospital operates in a healthcare environment, classified by UNDP as a low Human Development Index (HDI) country. Mulago Hospital is one of the main teaching hospitals in the countries surrounding Lake Victoria in East Africa. Mulago Hospital has a bed capacity of 1,500 patients, but the occupancy rate is around 200%.

Following approval by the research and ethics committee of Mulago Hospital an observational study was carried out among medical and paramedical staff for a period of three months from January to April 2007. The participants were purposively selected on the criterion of being any clinician, nurse or student who participates in the clinical process of blood transfusion. The study was carried out in the wards. Without interference with the normal routine in these locations, the participants were observed reaching conclusions on the diagnoses, taking decisions to transfuse, taking pre-transfusion samples, ordering for blood, transporting samples and request forms to the hospital blood bank and administering the blood to the patients. The staff members involved in blood transfusion were physicians, senior house officers, medical students, paramedical students, nurses and student nurses.

The observed patients (n = 41) were admitted at three different wards: the orthopedic ward, the Acute Care Unit for children and the gynecology and obstetrics ward. The observations were substantiated with two questionnaires. The first questionnaire was for collection of general information about the blood transfusion practice, like documentation, storage and transport conditions and the presence of guidelines. The second questionnaire was designed for the collection of patient data like history, symptoms, diagnosis, treatment plan and physical condition during interventions. The questionnaires were pre-tested for validity during the first week of the study by the observer and a senior doctor.

The observed staff members and patients were randomly chosen from those situations adhering to one strict criterion: the patient should receive a transfusion according to the treatment plan.

To enable comparison, a similar approach was followed in the University Medical Centre Groningen (UMCG), in the north of The Netherlands. This is a teaching hospital operating in a highly advanced health care environment in a high human development index country (high HDI). The hospital has 1,300 patient beds and has an occupancy rate of around 100%. The study in the UMCG was carried out in the oncology day care centre, orthopedic ward and traumatology ward, the departments that use blood most often in this institution.

**Figure 1:**
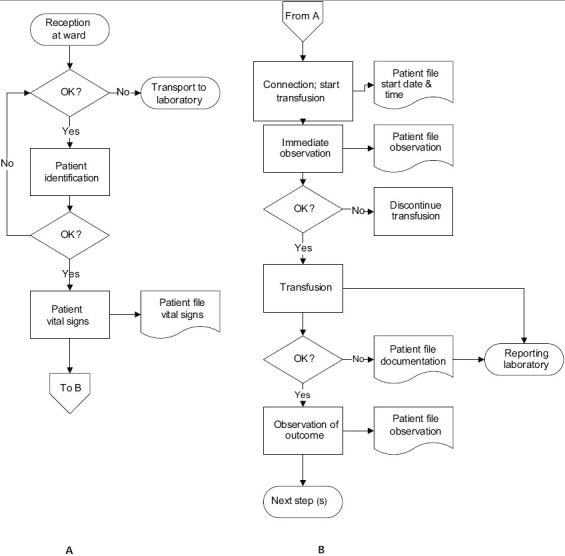
Flow chart for the reception of the blood unit on the ward (A) and the transfusion of the blood unit (B) (Smit Sibinga in: Popovsky 2007).[2]

The bedside blood transfusion practice, from the reception of the blood unit on the ward till the end of the blood transfusion chain, was analyzed following a specific flow chart [Figure 1].[2]

## Results

### Mulago Hospital

#### Guidelines

The current guidelines for blood transfusion published by Ministry of Health in Uganda were designed in 1989. These guidelines fall short in addressing key points in the bedside use of blood and put little emphasis on the creation and active participation of hospital transfusion committees in clinical use of blood. The Ministry of Health is currently designing new blood transfusion guidelines.

The current guidelines have not been well introduced and disseminated, and are not readily accessible on the wards in Mulago Hospital. Staff members, whether medical or paramedical, work with the knowledge and experience gathered during their education and on-the-job training. There is no uniformity and as a result staff members work on various professional levels. The absence of guidelines further enhances variation of practice. There is incomplete documentation, which seriously hampers traceability of data related to the transfusion episodes. So, there are no written process descriptions and hence no standard operating procedures to follow, describing the step-by-step order of the practice at the bedside.

#### Reception of the blood unit on the ward and patient identification

The units, which are issued by the hospital blood bank, are transported by hand to the ward by a ward or theatre staff member, e.g. a resident physician, nurse or student on duty on that ward. This staff member is also responsible for patient identification. Units are not stored or kept under appropriate conditions after arrival on the ward. Units selected for patients undergoing surgical interventions are kept in a domestic refrigerator. These are not appropriate for storage of blood since they do not have uniform temperatures and are not fitted with any control devices like audible alarms in case of temperature changes. These refrigerators run a serious danger of being contaminated with microorganisms, as cleaning and disinfection are not a common procedure.

On the wards the staff member will bring the unit to the patient without a formal identification procedure such as explicitly asking for name or date of birth of the patient. Patients do not wear a wristband with uniquely identifiable information, as these have not yet been introduced in Uganda.

#### Blood unit warming and patient vital signs

After the blood unit is received at the ward, it is warmed in the armpit of a relative for up to 30 minutes or in the bed of the patient for up to 3 hours. It is not a common practice for staff to record vital signs (temperature, pulse rate, blood pressure and respiration rate) before connection of the blood unit to the patient. This hampered interpretation of whatever symptoms developed during or after the transfusion. Hence, the root cause of a transfusion related reaction could not easily be traced.

#### Connection of the blood unit and immediate observation

The blood unit is connected by one of the medical staff members (either junior or senior member, whoever is involved). First a small bore needle or catheter is inserted in a vein and after that the infusion set is connected with the blood unit. A 0.9% sodium chloride drip is not used and the set is not rinsed before connecting to the unit of blood. This is done by all medical staff members and medical students, but medical students hardly know how to perform the procedure. These students learn without direct supervision or proper training, basically by trial and error. Standard principles of asepsis are poorly and inconsistently observed, as is the position of the needle in the vein. There were episodes observed where a needle or a connection set was not available and the transfusion had to be postponed. Meanwhile, a relative was urged to buy a needle or a giving set on the market.

There is no immediate and continuous observation of the patient. Therefore transfusion reactions and obstructions of the blood transfusion system and flow were not constantly observed. At certain points during the study it was observed in the department of Pediatrics that a unit of blood was shared among two or three children and ran simultaneously or in succession.

#### Transfusion and observation of the outcome

During the transfusion there is no observation of the patient. After the transfusion, there is no observation and recording of the outcome. However, bedside observations of transfusions were noted in emergency cases and as a component of the routine patient observations during the daily or sometimes only weekly ward rounds.

#### Documentation in the patient file

The documentation of the transfusion is very limited; of all transfusions observed around 50% were documented in the patient file, but only briefly and inaccurately. The documentation consisted of the fact that a blood transfusion had been given, the volume of the given unit and the names and amounts of administered drugs prior to, concomitant with and/or post transfusion. The identification number of the blood unit, the starting time and duration of the procedure were documented occasionally, but not consistently. Cross match information and the pre and post transfusion vital signs were not recorded at all. The ultimate outcome of the transfusion was not documented in the patient file.

### University Medical Centre Groningen

#### Guidelines

According to the European Union (EU) Directive 2002/98/EC, all procedures for blood transfusion on the ward must be described and detailed in accessible guidelines.[3]The guidelines are available through the ICT network of the hospital and are followed by all medical staff members. All medical staff members have to prove their competence to perform the blood transfusion procedures. Without this proof, which is a test observed by an authorized senior staff member, they are not allowed to perform the blood transfusion procedure. So all medical staff members at UMCG work professionally and on the same level.

#### Reception of the blood unit on the ward and patient identification

Blood units are transported to the ward by a laboratory technician of the hospital blood bank carried by hand in a temperature-controlled environment (container) or by a fast tube post system. For acceptance of the blood unit one of the registered medical staff members has to sign the accompanying issue form. The identification number on the blood unit, the blood group and Rhesus type of the unit, the expiry date and the patient data are compared with the data on the issue form and with the data in the medical file of the patient. This is all done by two registered medical staff members (check and double check). Then the patient is identified with help of the bar coded patient identification wristband and by asking name and date of birth of the patient.

#### Blood unit warming and patient vital signs

In the UMCG a blood unit is warmed only in special indications and the warming is done using adequately controlled equipment. Before connection of the blood unit vital signs are measured and documented in the patient file.

#### Connection of the blood unit and immediate observation

The blood unit is connected by a registered medical staff member. First a large bore needle or catheter is inserted and thereafter the blood unit is connected. An infusion Y-set enables connection of the blood unit on one arm and a normal 0.9% sodium chloride solution on the other arm of the Y-set. So the blood transfusion system is rinsed easily and a saline drip could serve as a rescue in case of an acute adverse event or between two subsequent transfusions.

Ten minutes after the start of the transfusion the patient is observed for adverse transfusion reactions and the findings are documented in the patient file. During the observation patients are also frequently asked how they feel.

#### Transfusion and observation of the outcome

After the transfusion the patient is observed and asked about the physical condition. However, hardly any documentation of the outcome of the transfusion is done anymore in the nursing file. Post transfusion hemoglobin is measured and documented in the patient file. In the UMCG, subsequent bedside observations of the outcome are the immediate observations in case of specific acute health problems or the clinical observations during the daily ward rounds. There is specific evaluation of the outcome of the blood transfusions based on the requirements of the national hemovigilance system.[1]

#### Documentation in the patient file

The UMCG works with an advanced digital documentation system. All laboratory results, X-rays, medication requests and interventions are documented in a digital patient file, which is accessible to registered medical staff members managing the patient. Medical staff members are obliged to keep the file up to date. Besides the digital documentation system, every admitted patient has a nursing file, which contains all the written information about performed bedside procedures.

## Discussion

**Figure 2:**
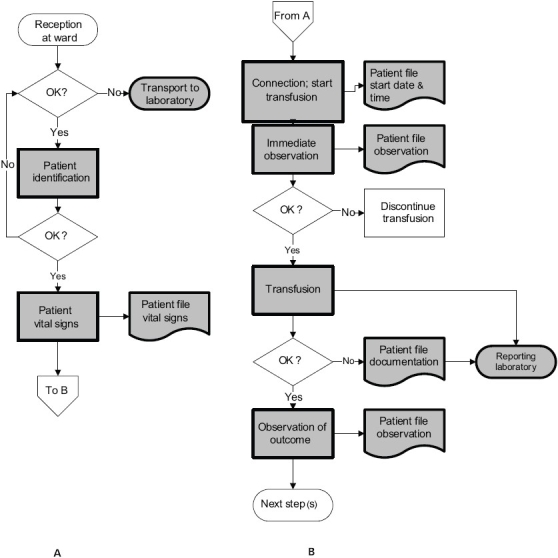
Flow chart for the reception of the blood unit on the ward (A) and the transfusion of the blood unit (B). The shaded symbols indicate the observed weaknesses in the bedside transfusion chain. (Smit Sibinga in: Popovsky 2007)[2]

The observational SWOT analysis revealed a series of weaknesses in the bedside transfusion chain of Mulago Hospital, which provide opportunities for improvement. The weaknesses observed in the bedside transfusion chain are marked in Figure 2. To enhance the quality of healthcare it is important that these weaknesses are identified and improved. However, it was observed that most staff involved seemed not really conscious of the importance of the work done, showing limited attention for both the unit of blood and the recipient of this precious gift of life.

### Reception of the blood unit on the ward and patient identification

Transportation of blood units in open air, over a length of time and distances in Mulago Hospital may cause warming of the blood unit due to the high ambient temperature. This temperature increase causes bacterial growth and hemolysis of the blood.[4,5]The environment of the UMCG, with a well functioning cold chain, ensures a controlled temperature for transportation of a unit of blood and guards against these iatrogenic compromises on blood safety. It should be noted, however, that the cold chain for preserving transport and storage conditions is not yet developed in Mulago Hospital and needs urgent attention from all stakeholders. Transportation in cool boxes and appropriate storage of the blood units on the ward or operation theatre are simple solutions to improve the quality of the blood units. The storage of unused blood under inappropriate conditions at Mulago Hospital also compromises the quality of the blood product. This can be followed by either bacterial or fungal growth in the blood bag, hemolysis, disturbed electrolyte balance, depletion of nutrients or accumulation of cell metabolites in the bag.

In Western countries 2/3 of the mistakes and errors made in the blood transfusion chain are identification mistakes.[6,7] Therefore it is important that the right blood unit is given to the right patient. This can be accomplished by an appropriate identification procedure. The WHO advises an identification procedure performed by two persons of whom at least one is a registered professional. During the identification process the name and date of birth of the patient must be asked, and the patient medical file and the identification wristband are checked.[8] Such identification protocol should be introduced and followed by a training program for the medical and nursing staff and the medical and nursing students. This should then be strengthened by regular audits. These WHO guidelines on patient identification are easily adhered to at the UMCG whose blood administering units are well staffed. On the other hand the units that use a lot of blood at Mulago Hospital like the department of pediatrics are poorly staffed and cannot afford to station two staff members to confirm identity of one patient for a given transfusion. This, however, can be mitigated by a well-designed training program of the pre-service and in-service staff in patient identification principles. This training should be given during education and later during special in-service trainings.

### Blood unit warming and patient vital signs

Blood units in the UMCG in The Netherlands are only warmed in specific indications, for example if the transfusion is intended for patients with clinically significant cold agglutinins. The warming is performed in specially designed equipment. In this way the blood is warmed before it enters the body, so there is little chance of blood contamination or hemolysis.[4,5,7] Keeping the patient warm is considered more important than warming the blood unit.

In Mulago Hospital all units are warmed, which is not necessary. The methods used to warm blood at Mulago Hospital, like placing it in the armpit of a relative or in the bed of the patient, increase the chances of bacterial contamination and damage (pinholes) to the plastic bag, hence compromising the safety of the transfused products in this institution.

To measure the outcome of a blood transfusion there is a need for reference points. These reference points are the vital signs of the patient and the patient’s hemoglobin level. Therefore vital signs of the patient should be measured before connection of the blood unit. It is difficult to interpret adverse transfusion reactions if the vital signs are not recorded prior to starting the blood transfusion. The measurement of reference points is an easy and inexpensive improvement of the blood transfusion practice, provided there is an adequate documentation system in place.

### Connection of the blood unit and immediate observation

In The Netherlands there is a special training program for medical students. The students are constantly supervised and they are not allowed to perform procedures by themselves. To increase the quality of healthcare, the procedures are performed by qualified medical or nursing staff members. In particular, blood units should be connected by registered medical staff members and medical students should constantly be supervised when doing this type of work. Blood as a tissue transplant predisposes recipients to adverse reactions, therefore it is paramount to observe transfused patients for adverse transfusion reactions. In the UMCG the blood transfusion protocol states that the patient be checked 10 minutes after starting the transfusion. In this way immediate adverse transfusion reactions can be observed. Thereafter the patient should be regularly observed.

In Mulago Hospital these vital steps of transfusion are overlooked. We postulate that this could be due to lack of prior training to the attending medical and nursing staff or due to the patient overload on the poorly staffed wards. This patient overload does not allow time for the attending medical staff to give the required supervision to the students as they perform bedside transfusion activities. The inadequate documentation of these baseline events undermines the interpretation of the subsequent transfusion observation. In the absence of adequate observation and documentation medical personnel do not react instantly or properly to any outcome. The hospital should therefore design a transfusion policy and strategy, which should include guidelines on immediate observations and documentation of a patient undergoing a transfusion. It is also important to provide appropriate training of nursing and medical staff on recognizing adverse transfusion reactions.

In addition to observing for adverse transfusion reactions, it is important to observe for the rate of flow of the blood. In Mulago Hospital, unlike at UMCG, flow rate of transfusions, especially of packed red cells, were observed to be interrupted on several occasions. This could have been due to the poor and inconsistent quality of transfusion sets and needles used, but could also be explained by the common un-attended positioning of the limbs with transfusion intravenous lines that cause kinks in the cannulas and infusion sets with subsequent interruptions in the blood flow. Besides, red cells (packed) in Uganda are very viscous because hardly any plasma or fluid is left on the cells during processing. Therefore more blood transfusions stop running. This obstruction of the flow problem should be resolved as soon as possible to facilitate an optimal effect of the treatment.

### Transfusion and observation of the outcome

Observation of the outcome of a blood transfusion is a developing process in Western countries, yet largely absent in developing countries. For the remaining treatment of the patient it is important to directly observe the outcome of the blood transfusion.

### Documentation in the patient file

For the recovery process of the patient it is paramount to document all critical steps. If something goes wrong all documented steps can be traced and analyzed in the patient file. In The Netherlands documentation is regulated by law and therefore always will be done. Additionally, in an increasing number of hospitals electronic patient files have been introduced.

In Mulago Hospital there is little documentation and this is mostly poorly legible. The quality of the patient file is also poor, so papers or documents are easily lost. To improve the quality of healthcare, and blood transfusion as an integral part, much improvement needs to be made by developing an appropriate documentation system.

### Guidelines

In the absence of available and/or accessible hospital guidelines for blood transfusion it is hard to teach medical students appropriately. In most Western countries policies and guidelines are available and accessible. These guidelines should contain various aspects, which are mentioned in the guidelines for blood transfusion developed by WHO.[8] Therefore a policy should be developed for blood transfusion together with appropriate guidelines.

### Conclusion and recommendation

In a developing world situation, like Mulago Hospital, Kampala, Uganda

- the blood transfusion practice on the ward is far from optimal.
- the blood transfusion practice is likely to play a role in morbidity and mortality of patients who receive a blood transfusion.

To improve the blood transfusion practice there is need for a blood transfusion policy and strategy, including appropriate guidelines. After the introduction of such policy it is important to provide training to medical and nursing staff, as well as to students and laboratory staff. It is crucial that medical students are constantly supervised during their internship.

To show the importance of a well-developed blood transfusion policy there is a need to clearly present the consequence of a less developed blood transfusion practice. In particular, adverse transfusion reactions should be observed accurately, documented and evaluated. Subsequently this may serve as a strong rationale to improve the blood transfusion system. In the end this will help to adequately motivate the professionals to support the development and introduction of formal transfusion policies and guidelines, and to adequately adhere to the latter.

Comparison with the blood transfusion policy of the UMCG gives an indication of a well-developed blood transfusion chain. However, the blood transfusion policies of the two hospitals are so different, that it can only be concluded that a well-planned step-by-step improvement in Mulago Hospital is needed, where the UMCG could serve as a reference.

The improvement of the clinical blood transfusion practice in Mulago Hospital will be a challenging task, because of the economic situation and the mindset in Uganda. There is little money available for the organization of seminars, courses and for the organization of a blood transfusion committee. There are also limited resources available to improve the patient files and to introduce proper patient identification through the use of wristbands.

With the help of international professionals in the field of transfusion medicine it might be possible to improve the blood transfusion practice in some aspects in the near future and observed adverse reactions could be treated in a timely and effective manner. However, structured organization and infrastructure as well as the implementation of quality principles in the hospital and a change in the mindset are paramount to such development.

## References

[1] TRIP 2006. TRIP report 2006.

[2] Smit Sibinga CTh, Popovsky MA (2007). Detecting and monitoring reactions in the developing world. Transfusion reactions.

[3] Directive 2002/98/EC (2003). Official Journal of the European Union.

[4] Williamson JR, Shanahan MO, Hochmuth RM (1975). The influences of temperature on red cell deformability. Blood.

[5] Fischer TM (2004). Shape memory of human red blood cells. Biophys J.

[6] Pagliaro P, Rebulla P (2006). Transfusion recipient identification. Vox Sang.

[7] Serious Hazards of Transfusion, annual report 2005. The Serious Hazards of transfusion Steering group.

[8] (2005). The Clinical Use of Blood. Handbook.

